# Exploring the Polymorphism of Drostanolone Propionate

**DOI:** 10.3390/molecules25061436

**Published:** 2020-03-21

**Authors:** Gheorghe Borodi, Alexandru Turza, Attila Bende

**Affiliations:** 1 National Institute For R&D of Isotopic and Molecular Technologies, 67-103 Donat, Cluj-Napoca 400293, Romania; borodi@itim-cj.ro; 2 Faculty of Physics, Babeş-Bolyai University, 1 Mihail Kogălniceanu, Cluj-Napoca 400084, Romania

**Keywords:** drostanolone propionate, steroids, crystal structure, X-ray diffraction, Hirshfeld analysis, lattice energy

## Abstract

2α-Methyl-4,5α-dihydrotestosterone 17β-propionate, known as drostanolone propionate or masteron, is a synthetic anabolic-androgenic steroid derived from dihydrotestosterone. The crystal structures of two polymorphs of drostanolone propionate have been determined by single crystal X-ray diffraction and both crystallizes in the monoclinic crystal system. One is belonging to the P2_1_ space group, Z = 2, and has one molecule in the asymmetric unit while the second belongs to the I2 space group, Z = 4, and contains two molecules in the asymmetric unit. Another polymorph has been investigated by an X-ray powder diffraction method and solved by Parallel tempering/Monte Carlo technique and refined with the Rietveld method. This polymorph crystallizes in the orthorhombic P2_1_2_1_2_1_ space group, Z = 4 having one molecule in the asymmetric unit. The structural configuration analysis shows that the A, B, and C steroid rings exist as chair geometry, while ring D adopts a C13 distorted envelope configuration in all structures. For all polymorphs, the lattice energy has been computed by CLP (Coulomb-London-Pauli), and tight-binding density functional theory methods. Local electron correlation methods were used to estimate the role of electron correlation in the magnitude of the dimer energies. The nature of the intermolecular interactions has been analyzed by the SAPT0 energy decomposition methods as well as by Hirshfeld surfaces.

## 1. Introduction

The polymorphism in organic compounds is the ability to exist in several crystalline forms. The polymorphs have similar chemical content but exhibit different crystal packing and arrangement [1]. Androgens represent a class of compounds, which can be synthetic or naturally found in vertebrates that are responsible for development and maintenance of male characteristics. The anabolic component of a certain anabolic-androgenic steroid is related to increased protein synthesis in muscle tissues and bones, while the androgenic component is responsible for the development of secondary male characteristics such as deepening of the voice, facial and body hair growth, and aggressiveness. Synthetic androgens are chemically-modified forms of the primary male hormone, testosterone, with the purpose to lower the androgenic characteristics and increase the anabolic properties [2]. Despite the fact that anabolic-androgenic steroids fulfill certain functions in vertebrates and are very effective to boost sports performance, their misuse and abuse can lead to undesirable, serious negative side effects on health. Heart diseases (hypertension, left ventricular hypertrophy, impaired diastolic filling, polycythemia, and thrombosis) are known to be related to the long-term consumption of anabolic steroids [3]. A decrease in the high density lipoprotein (HDL), concomitant with an increase in a low density lipoprotein (LDL) and total cholesterol, are related to the consumption as well, which may increase the risk of atherosclerosis in the coronary arteries [4,5,6]. Hypogonadism induced by anabolic steroids in males and the development of male characteristics in women and children are regarded as having milder side effects and they are reversible to some extent [7]. Besides the anabolic properties of the derivatives in this group, they can be useful in certain medical conditions, which cause undesired catabolism and loss of muscle mass. They act as anti-catabolic, which negates the effects of cortisol and the derivatives from glucocorticoids class [7,8]. 

**Figure 1:**
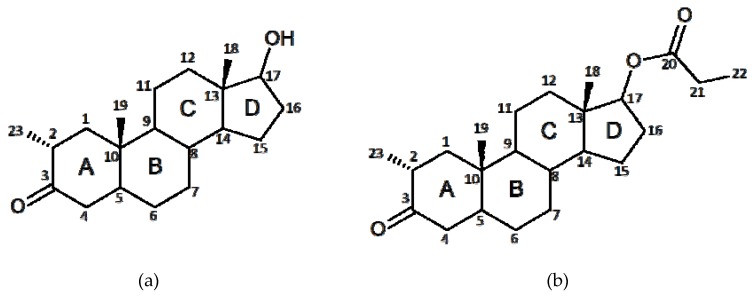
Chemical configurations of (**a**) drostanolone and (**b**) drostanolone propionate displaying the atom labeling system.

Drostanolone propionate (2α-Methyl-4,5α-dihydrotestosterone 17β-propionate), known with the trade name as masteron (Figure 1b), is an androstane steroid, derived from dihydrotestosterone (Figure 1a) [9,10]. The scheme of atoms and rings labeling was made according to the established notations of the compounds in this class [9]. It works in the same manner as any androgenic steroid, being an agonist of the androgen receptor [11]. Medically, it was used in breast cancer treatment [9] and, in addition, is often used in sport, bodybuilding, and powerlifting as a performance enhancer, while providing increased protein synthesis, which reflects in the gain of lean muscle mass tissue and aids recovery [9]. Often anabolic-androgenic steroids are esterified with the intention to increase the duration of action by intramuscular or subcutaneous administration [12,13]. Drostanolone propionate is an injectable steroid, which is modified by esterification of the parent hormone (drostanolone) in the position of the O-H hydroxyl group (Figure 1). Several esterized forms of drostanolone are available on the market. These include drostanolone propionate that has been investigated for treating breast cancer [14], drostanolone enanthate, which by microbial transformation, led to the synthesis of eight potentially anti-cancer metabolites [15]. Since there are no studies regarding the crystal structure of drostanolone propionate and its polymorphism, this paper will focus on the structural aspects of this anabolic-androgenic steroid. For the starting compound (denoted Drost 1), no single crystals were obtained and the structure was solved from powder diffraction data. Using samples from the starting compound (Drost 1), by recrystallization in ethanol, the polymorph Drost 2 was obtained and, by recrystallization of the starting compound in acetone, was obtained by polymorph Drost 3.

## 2. Results and Discussion

**Figure 2:**
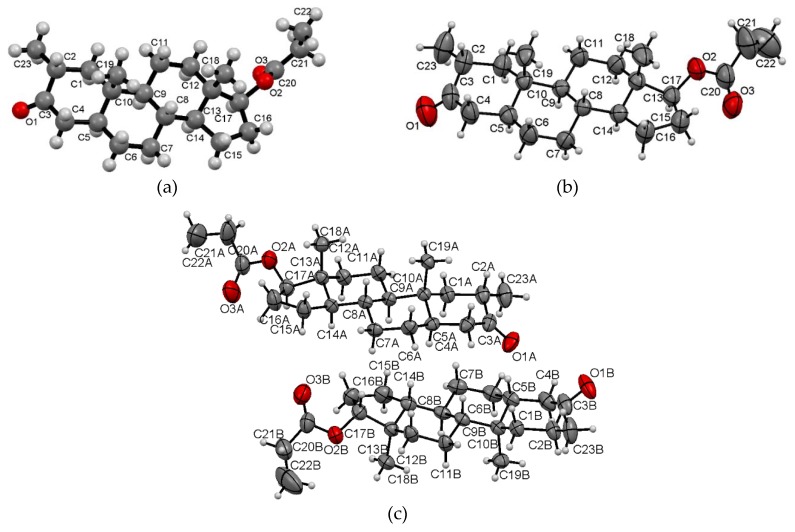
Molecular configuration of the asymmetric units for Drost 1 (**a**), Drost 2 (**b**), and Drost 3 (**c**). Drost 2 displays the thermal ellipsoids at 50% probability and Drost 3 at 30% probability level.

The chemical configurations of drostanolone and drostanolone propionate are depicted in Figure 1, while overall conformations of the molecules in the asymmetric unit are shown in Figure 2. Since, in Drost 3, there are two molecules in the asymmetric unit (denoted with molecule A and molecule B), in Figure 2c, both molecules are presented. The most important crystallographic data are collected in Table 1. 

### 2.1. Crystal Structure Analysis

**Figure 3:**
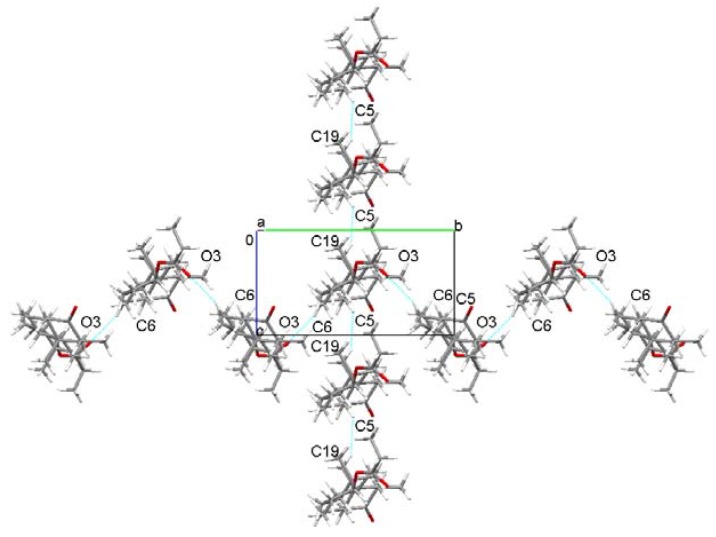
Packing diagram of Drost 1 showing the molecular chains in ob and oc directions.

Figure 3 presents the molecular packing of Drost 1, viewed along the a-axis direction. The molecules of Drost 1 are linked by C6-H...O3 contacts formed between the carbonyl O3 oxygen and C6 carbon belonging to the B steroid ring and are building molecular sinusoidal chains in the direction of the b-axis. Along the c-axis, the molecules are linked by C5-H...H-C19 short contacts formed between C5 carbon, which belongs both to A and B rings toward the C19 methyl group. This pattern is repeating by translations equal to lattice parameters both along the c-axis and b-axis.

**Figure 4:**
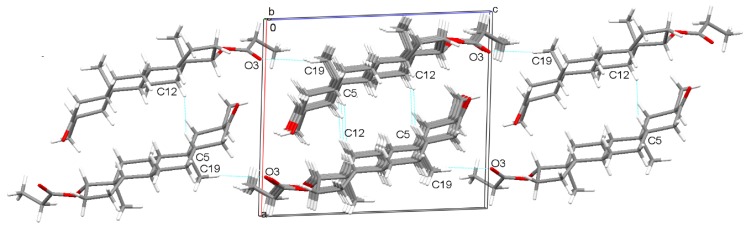
Unit cell packing diagram illustrating the arrangement of Drost 2 molecules.

The unit cell of Drost 2 contains two molecules related by a 2_1_ screw axis linked by C5-H...H-C12 short contact, formed between C5 carbon, which belongs to both A and B steroid rings and C12 carbon of the C ring. The pattern is further extended by translations and forms an arrangement parallel to the ob direction. At the same time, along the c-axis, an infinite arrangement of molecules is formed, which are linked between O3 carbonyl oxygen and C19 methyl group by C19-H...O3 hydrogen bond, as shown in Figure 4.

**Figure 5:**
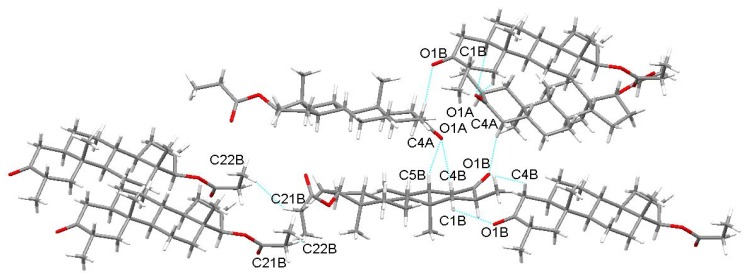
Short contact interactions in Drost 3.

The unit cell of Drost 3 fits eight drostanolone propionate molecules. The two independent molecules in the asymmetric unit of Drost 3 are held by bifurcated C5B-H...O1A and C1B-H...O1A hydrogen bonds between the carbonyl O1A oxygen with C1B and C5B carbon atoms of the A ring in molecule B. The C4A-H…O1B hydrogen bond links the C4A carbon of molecule A with the carbonyl O1B oxygen. Within the structure, the B molecules are held together by a combination of C4B-H…O1B between C4B carbon of the ring A with the oxygen O1B and the C21B-H…H-C22B short contact interaction extended between C21B and C22B carbons of the propionate ester (Figure 5). Mercury software [16] was used to generate perspective molecular views and the packing diagrams.

### 2.2. Crystal Structure Determination of Drost 1 by X-ray Powder Diffraction (XRPD)

The crystallization attempts to obtain suitable single crystals of Drost 1 have failed and it was needed to undertake the crystal structure determination by the XRPD method. This is a multi-step method and involves the following: the X-ray pattern indexing, Pawley refinement, space group assignment, structural model determination, and Rietveld refinement [17].

The pattern indexing was accomplished in the Reflex module, which is implemented in Materials Studio software [18]. Analyzing the unit cell solutions and based on the figure of merit (F.O.M), an orthorhombic common solution was obtained by using four different programs: DICVOL96 [19], TREOR90 [20], ITO15 [21], and X-cell [22]. Taking into account the first 30 lines, a very high FOM is obtained as follows: FOM = 50 with TREOR90, FOM = 49 with DICVOL96, FOM = 9.7 with X-cell, while with ITO15, the FOM = 73 was obtained by indexing the first 20 diffraction lines. The orthorhombic solution (a = 27.2543 Å, b = 12.078 Å, c = 6.4178 Å) has successfully indexed all selected lines. 

Pawley refinement procedure (R_wp_ = 4.02%) has confirmed that the crystal system is orthorhombic P2_1_2_1_2_1_ as being the most likely space group with one molecule of drostanolone propionate in the asymmetric unit.

As a starting point for finding the structural model, the CIF (Crystallographic Information File) file of the Drost 2 polymorph was obtained by single crystal X-ray diffraction in order to have the most reasonable bond distances. The model was optimized by altering torsion angles translations and, respectively, rotations. A single molecule was considered in the asymmetric unit because the calculated density using the adopted spatial group is 1.14 g/cm^3^, which is a reasonable value considering the chemical composition of the compound. The structural solution model was determined by using direct-space Monte-Carlo with parallel tempering methods in Fox software [23]. This is a trial and error method, which works by comparison between experimental and calculated patterns and, when they are in agreement, the solution is subjected to the Rietveld refinement. 

**Figure 6:**
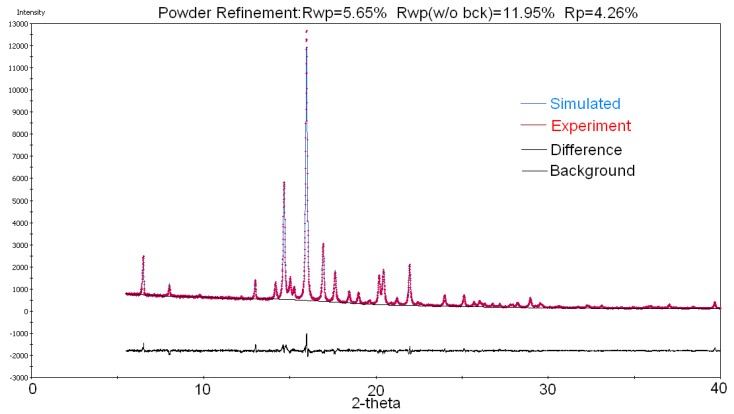
Rietveld refinement and the agreement between simulated and experimental patterns.

In the Rietveld procedure, which was carried out with a Reflex module from Materials Studio software, was included as the refinement of the following parameters: diffraction peaks, which were approximated as Pseudo-Voight function, (U, V, W) parameters from the Caglioti formula [24], profile parameters NA, NB in Bragg-Brentano geometry, zero point shift parameter, peaks asymmetry parameters from Berar-Baldinozzi approximation (P1, P2, P3, P4), the parameters that describe preferred orientation (a^*^, b^*^, c^*^ and R0) in March-Dollase correction, and the coefficients describing the background profile. The final fit between calculated and measured patterns shows a good agreement, with R_wp_ = 5.65% (Table 2 and Figure 6).

### 2.3. Hirshfeld Surfaces and Fingerprint Plot Analysis

#### 2.3.1. Hirshfeld Surfaces

**Figure 7:**
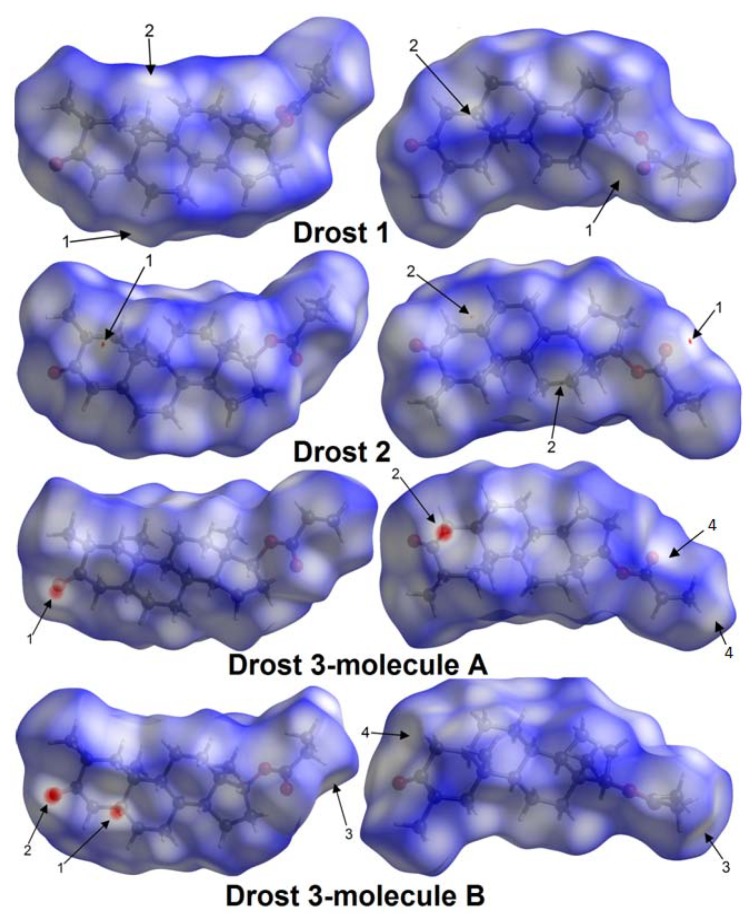
Hirshfeld surfaces showing the interactions shorter than van der Waals radii with the contacts referred to Table 3. The color scale for the d_norm_ property mapped on the Hirshfeld surface is in the range of 0.013 (white) to 1.563 (blue) for Drost 1, −0.016 (red) to 1.444 (blue) for Drost 2, −0.151 (red) to 1.628 (blue) for Drost 3.

Front views and back views of the three-dimensional Hirshfeld surfaces (mapped with d_norm_) for studied drostanolone propionate structures are given in Figure 7. The contacts mapped in red represent the interactions with the distances between atoms smaller than the sum of van der Waals radii, white areas highlight intermolecular contacts close to the sum of van der Waals radii, and blue is used for longer contacts [25]. The Hirshfeld surfaces were mapped with d_norm_ between −0.151 (red) and 1.628 (blue). Figure 7 shows the Hirshfeld surfaces’ front views, respectively, back views of each polymorph, and intermolecular interactions smaller than the sum of van der Waals radii showed as labelled arrows. Each label illustrated on the Hirshfeld surface has the interaction geometry detailed in Table 3.

**Figure 8:**
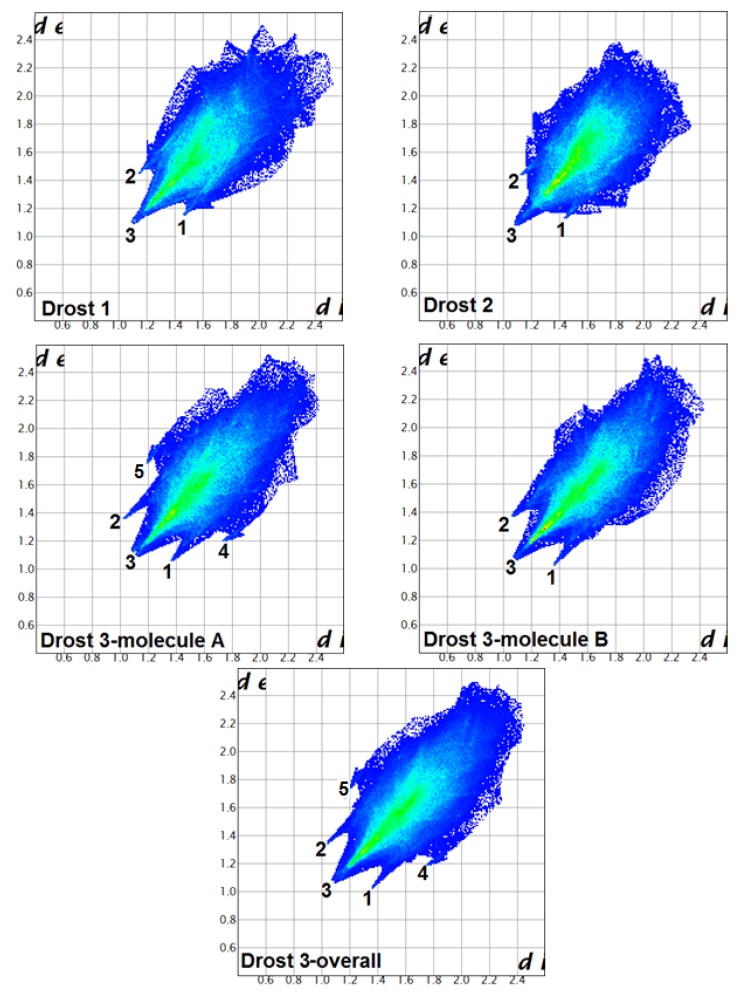
Fingerprint plots for studied compounds showing labelled close contacts O...H (1), H...O (2) and H...H (3), C…H (4), H…C (5).

Figure 8 illustrated the fingerprint plots of the 3D Hirshfeld surfaces for Drostanolone propionate polymorphs. The evaluation of fingerprint plots provide the division of different contributions for H...H (labelled as 3), O...H (labelled as 1), H...O (labelled as 2), C…H (labelled as 4), and H…C (labelled as 5) interactions.

In Table 4, the intermolecular contacts X...H/H...X, (where X can be O or C). The first letter signifies the atom located inside the Hirshfeld surface and the second signifies the atom outside the surface.

The fingerprint plot of Drost 1 presents small spikes (labelled as 1 and 2), which are related to O…H/H…O interactions.

Spike 2 is caused by the C6-H6A donor group, which is located inside the Hirshfeld surface and the O3 acceptor of the carboxyl group is situated outside the surface. The contact is taking place at distance d_i_ + d_e_~2.6 Å. The complementary spike 1 accounts for the same interaction but the O3 acceptor is inside the surface and the donor outside. The spike labelled as 1 is related to H...H interactions, which extends to the smallest distance of d_i_ + d_e_~2.2 Å with the hydrogens being inside and outside the surface as well.

The fingerprint plot of Drost 2 show similar spikes (labelled as 1 and 2) as Drost 1, which are characteristic to O…H/H…O inter-contacts. The spike denoted by 2 indicates the C19-H19B donor, which is situated inside the Hirshfeld surface and connects with O3 carboxyl outside the surface. The intercontact distance extends to d_i_ + d_e_~2.57 Å. Spike 1 is complementary with spike 2, but the O3 carboxyl is inside the surface, while the donor is situated outside. The H...H interactions, displayed as label 1 on fingerprint plot, are characterized by the sum of d_i_ + d_e_~2.17 Å.

#### 2.3.2. Fingerprint Plot Analysis

The fingerprint plots for molecule A and molecule B in Drost 3 exhibit roughly the same overall d_i_ and d_e_ range. The A molecule is characterized by two wings (labelled as 4 and 5), which is related to C...H/H...C inter-contacts and a wide H...H spike (labelled as 3), which tends to be slightly split. By contrast, molecule B lacks these two features buts shows similar O...H/H...O and H...H spikes. The O...H/H...O inter-contacts extends at the lowest distance d_i_ + d_e_~2.4 Å (molecule A) with the C4A-H4AA donor situated inside the Hirshfeld surface and O1B of the carboxyl group exterior to the surface and d_i_ + d_e_~2.44 Å, where the donor is C5B-H5B inside the surface and the acceptor O1A outside the surface (molecule B). In previous compounds, the spike labelled by 2 is complementary with spike 1. H...H contacts extend toward the lowest intercontact distance d_i_ + d_e_~2.2 Å (molecule A) and d_i_ + d_e_~2.16 Å (molecule B). The spikes 4 and 5, which stand for C...H/H...C interactions, are present only in the A molecule of Drost 3, which have the distance d_i_ + d_e_~2.95 Å with the C22A-H22C donor inside the surface and C20A carbon outside. Considering the Hirshfeld surface generated for an overall structure of Drost 3, the contacts (between the two molecules in the asymmetric unit) C5B-H5B...O1A (label 1, Figure 8), C1B-H1BB...O1A (label 1, Figure 8) and C4A-H4AA...O1B (label 2, Figure 7) would disappear. Meanwhile, the corresponding fingerprint plot resembles the fingerprint plot of molecule A.

The fingerprint breakdown shows that, in all three crystals, the H...H interactions have the higher participation with respect to the other contacts, the O...H/H...O interactions represent the second molecular contact by participation C...H/H...C intercontact becomes much less significant. The percentage contributions to the Hirshfeld surfaces areas for studied crystals are represented in Table 4. The conclusions resulting from fingerprint plots analysis include the following.

(i) The plots shape and features are different in all three compounds and indicate that the supramolecular assemblies are different for each crystal structure.
(ii) The top end values of d_e_ and d_i_ in the fingerprint plots of Drost 2 are slightly smaller in comparison with Drost 1 and the two independent molecules of Drost 3, which conclude that Drost 2 has higher packing efficiency [26]. This is already in good agreement with the Kitaigorodskii packing index.
(iii) The fingerprint plots of Drost 1 and Drost 2 exhibits similar features, while being different with the plots of the two molecules in Drost 3.
(iv) The common and the most visible feature in all polymorphs is the wide H...H spike, which stands for label 3.
(v) The two distinct molecules of Drost 3 show two sharp spikes, which are characteristic to O...H and H...O contacts. These are less protruding in Drost 1 and Drost 2, which indicate the formation of stronger C-H…O bonds in Drost 3.
(vi) The C-H…C interactions in molecule A of Drost 3 are seen on the fingerprint plot as two characteristic wings, which molecule B is lacking.
(vii) The high percentage of H…H, O…H and C…H inter-contacts indicates that the structures rely on weak van der Walls interactions, which assure the crystal packing [27].

### 2.4. Lattice Energies Evaluation

#### 2.4.1. Lattice Energy Evaluation by the Coulomb-London-Pauli (CLP) Method

The CLP method (See details at Section 3.6), which is based on atom-atom type potentials, has been shown that the formation of two drostanolone propionate polymorphs has led to structures that display similar lattice energies (−156.3 kJ/mol in Drost 1; −159.2 kJ/mol in Drost 2 and −151.6 kJ/mol in Drost 3, respectively).

The partitioned packing energies calculated for the polymorphs driven by slow evaporation in ethanol (Drost 2) and acetone (Drost3) shows similar values as the start compound (Drost 1). The total lattice energy breakdown in individual terms is given in Table 5a. The electrostatic terms, which account for Coulombic and polarization term have a small contribution, while the dispersion energy plays the major role. Specific interatomic interactions that contribute mainly into the total intermolecular interaction energy are given in Table 3. Although the O...H contacts have a lower preponderance than the H...H contacts (Table 4), they have a high energy and contribute in the greatest extent to lattice energy. 

Similar behavior regarding lattice stability through weak H...H and O...H intermolecular interactions was found in compounds of corticosteroid class [28]. Such compounds were investigated by adsorption and Raman scattering, which is assigned to intermolecular interaction and specific effects of crystal packing by comparing experimental spectra with quantum chemical calculations [29].

The Kitaigorodskii packing index [30] is a measure of packing efficiency and usually has a value of 65%. It was evaluated by PLATON software [31] and the results obtained are as follows: 60.13% for Drost 3, 60.25% for Drost 1, and the highest value of 61.64% for Drost 2. Between the packing index and total CLP lattice energy, there is a correlation. The higher the packing index is, the greater the absolute value of the lattice energy is. From Table 5a, it is observed that this correlation between packing index and lattice energy is noticed.

#### 2.4.2. Lattice Energy Evaluation by a Density-Functional Tight-Binding Model

In order to validate our method based on the atom-atom potential-type CLP model, a higher level theoretical method was also considered. Accordingly, the density-functional tight-binding (DFTB) model in its self-consistent charge corrected the variant (SCC-DFTB) [32] implemented in the DFTB+ code [33] and applied in order to compute lattice energies for the three crystal configurations [34]. Several supercells with different cell sizes (1 × 1 × 1, 1 × 1 × 2, 1 × 2 × 2, 2 × 2 × 2, 2 × 2 × 3, 2 × 3 × 3, 3 × 3 × 3) were generated [35] and their electronic energies were computed, including the dispersion effects via the Slater-Kirkwood (SK) dispersion correction scheme [36]. Based on our past experience [37], compared with results computed using the second order Møller-Plesset perturbation theory, the SK-dispersion model can well reproduce the dispersion effects in the case of large molecular clusters. The largest supercell structure (3 × 3 × 3) for Drost 2 crystal conformation includes 87 monomers (5394 atoms) while those for Drost 1 and Drost 3 (2 × 3 × 3) contain 134 (8308 atoms) and 164 monomers (10168 atoms), respectively. Then, the total electronic energy values of the supercells with different cell sizes defined by the number of monomers in the supercell were extrapolated into the infinite large monomer numbers and the lattice energy, as the size independent parameter in the interpolation scheme (parameter *a*), was obtained [34]. The best performant fitting function (R^2^ ≈ 0.94) was found as:

(1) $$f\left(N\right)=E^{latt}\left(N^{\infty }\right)+\frac{a}{\sqrt[{4}]{N}}$$

where *N* is the number of the unit cells, *a* is the fitting parameter, and *E^latt^* means the lattice energy for an infinite number of unit cells. 

The dispersion component of the lattice energy considering the same interpolation scheme (See Equation (1)) was also computed. Accordingly, the lattice total energies and their dispersion parts computed for the three unit cell configurations with the above described calculation scheme are presented in Table 5b. Comparing the results obtained based on the CLP model [38,39] and by SCC-DFTB theory, relatively good agreement between the two theories can be found for the lattice energy values. This agreement looks effective for the case of Drost 2 crystal configuration (the energy difference is 7.5 kJ/mol), while, for the Drost 1 and Drost 3, the energy deviations are a bit larger: 13.7 and 13.9 kJ/mol, respectively. Furthermore, both theoretical methods give the Drost 2 conformation as the most bounded crystal structure followed by Drost 1, while the Drost 3 crystal has the smallest cohesive energy value. Furthermore, both methods suggest that the main attractive forces, which keep the crystalline structures together are the dispersion effects. 

**Figure 9:**
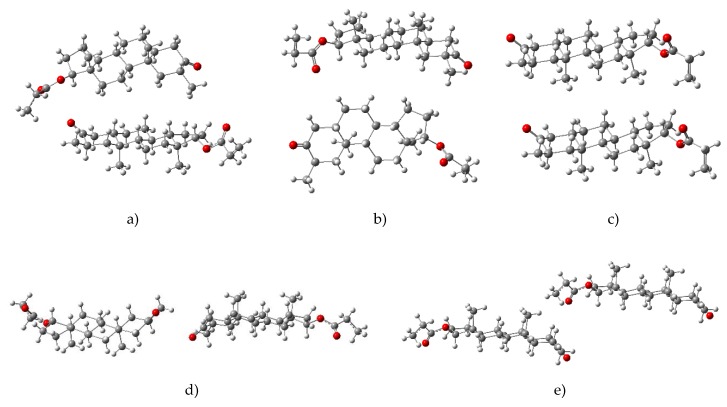
The geometry configurations of the five dimer geometries characteristic for the Drost 2 unit cell’s close contacts. The dimer geometries are indexed from **a**)–**e**).

In order to better understand the nature of the intermolecular forces, which govern the formation of the molecular crystals, higher level electronic structure theory was also considered. Accordingly, the molecular dimer energies for different dimer geometry conformations taken from the Drost 2 crystal supercell were computed using the second order Møller-Plesset perturbation theory based on localized molecular orbitals and density-fitting technique (DF-LMP2) [40]. The local correlation treatment also offers the possibility to decompose the intermolecular interaction energy into intramolecular, dispersive, and ionic components of the correlation contribution. Five different dimer geometries, relevant for the unit cell configuration, were selected (see Figure 9). The selection criterium is based on the Drost 2 unit cell configuration, where all close contacts (defined as the sum of the vdW radii + 0.2 Å) of the Drost 2 molecule from the unit cell were generated and, from the resulted oligomer cluster, all the possible dimer configurations were kept.

The intermolecular interaction energies between the drostanolone propionate monomers were computed considering the DF-LMP2 method and using the def2-tzvp [41] basis set, as implemented in the Molpro program package [42]. Binding energies were estimated in the supramolecular approximation (i.e., as a difference between the energy of a given dimer complex and the sum of energies of the isolated molecules constituting it). The Drost 2 crystal structure is built by parallel layers grown in the aob crystal plane, where layers are kept together by the side-chain interactions of the drostanolone molecules along the oc crystal axis. Three dimer configurations (see Figure 9a)–c)) were identified as relevant pair conformations for the layer structure stability (parallel stacking, T-shape, and antiparallel stacking). Their intermolecular interaction energies obtained at the DF-LMP2/def2-tzvp level of theory are: ΔE^a)^ = −21.67 kJ/mol, ΔE^b)^ = −15.37 kJ/mol and ΔE^c)^ = −18.08 kJ/mol, respectively. On the other hand, the interaction between the layers is mainly built by two characteristic pair configurations (See Figure 9d,e). Their intermolecular interaction energies computed at DF-LMP2/def2-tzvp level of theory are: ΔE^d)^ = −8.25 kJ/mol and ΔE^e)^ = −14.13 kJ/mol, respectively. To estimate the contribution of higher (other than pair correlation) electron correlation effects to the intermolecular interaction energy, the coupled cluster level of theory based on localized molecular orbitals and density-fitting technique (DF-LCCSD(T)) for the a) dimer configuration was considered. The result shows that higher level electron correlation effects do not change the magnitude of the intermolecular interaction energy significantly, which gives only a small contribution (+1.18 kJ/mol) to the final energy value (−21.67 kJ/mol for DF-LMP2 versus −20.49 kJ/mol for DF-LCCSD(T)). Since advanced ab initio theories including high-level electron correlation effects are not suitable to directly compute the lattice energies due to the large amount of computation, they can be used indirectly to estimate the lattice energy as a sum of the energies of intermolecular (noncovalent) pairwise interactions between the considered molecule and its neighbors [43]. Accordingly, in the case of the Drost 2 molecule, the total pairwise interaction energy between a monomer from the crystal and its neighbors (close contacts) can be computed as the sum of the binding energies of the five dimers but consider each of them twice, since in the close contact configuration, each of them occurs two times. In this way, the sum of the pairwise interaction energies is −155.00 kcal/mol, which is consistent with previous lattice energy calculations for the Drost 2 case based on the CLP and SCC-DFTB methods (−159.2 kcal/mol and −151.7 kcal/mol, respectively). On the other hand, the Espinoza’s empirical formula [44] defined in the framework of QTAIM (or quantum theory of atoms in molecules) theory [45] can be used as another possible solution for estimating the lattice energy. This solution has been successfully applied in several cases [46,47,48] based on Density Functional Theory calculations, but applying it in the case of the DF-LMP2 theory is not a simple task. 

Using the CLP and the SCC-DFTB simple models, only the global contribution of the dispersion effects was taken into account. However, it would be interesting to analyze how the dispersion effects manifest along the different crystal axes, since it is shown in Figure 4 that different relative pair conformations can be observed along the oa and ob or oc axes. More precisely, the binding energies of dimers a)–c) build layers along the oab crystal plane, while interactions found in case of dimers d) and e) enhance the crystal cohesion along the oc direction. Accordingly, for the five dimer cases of the Drost 2 crystal configuration, the symmetry adapted perturbation theory (SAPT) method [49] was applied to decompose their intermolecular interaction energy into physically meaningful energy components (electrostatic, exchange, induction, and dispersion) defined in the framework of the SAPT theory. Using the SAPT energy decomposition method, our goal was to get rather realistic values than to directly compare them with results obtained by other theoretical methods, like DF-LMP2. Due to the large numbers of atoms in the dimer geometry, applying the so-called “gold” and “silver” standards for the SAPT method are not feasible. Only the “bronze” standard is feasible. This standard is defined as the zero-order SAPT expansion combined with the exchange-scaling (sSAPT0) approximation [50] and used together with the jun-cc-pVDZ basis set [51]. The intermolecular energy values and their different energy components computed at sSAPT0 as well as the intermolecular energy values computed at DF-LMP2 levels of theory are presented in Table 6. 

In the case of dimer configurations having parallel stacking or a), T-shape or b) and antiparallel stacking or c) conformations, the most dominant energy component is the dispersion part, which is almost equal or even lower than the total sSAPT0 energy, but it is almost half balanced by the exchange repulsion effects. The electrostatic and induction contributions individually have a relatively small contribution to the sSAPT0 energy, but their common effect is no longer a negligible contribution. In the case of layer-layer interaction, which includes the d) and e) dimer configurations, in addition to dispersion and exchange interactions, electrostatic contributions have also become important. All these results show us that the layer structure defined by the a)–c) interaction configurations (oa and ob crystal axes directions) are much stronger bound than the interaction between the layers (along the oc crystal axis). 

### 2.5. Conformational Analysis

The A, B and C rings of the steroid skeletons were found as chair conformation, whereas D rings are adopting a C13 envelope conformation in all three structures. 

**Figure 10:**
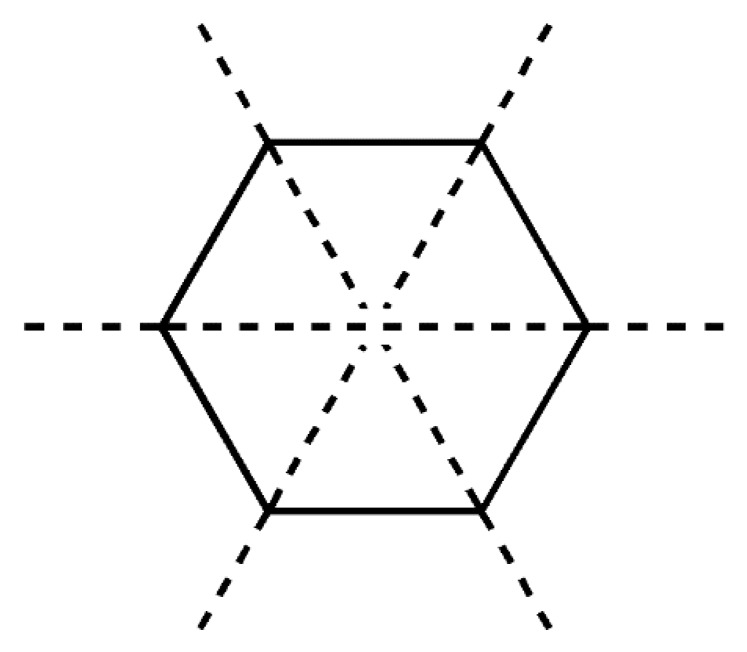
Pseudo mirror planes related to ΔC_s_.

The displacement in the ring’s geometry from the ideal chair conformation was described by the asymmetry parameter ΔC_s_ evaluation [52]. The calculated values of the asymmetry parameter ΔC_s_ show that the geometry of the A, B, and C rings for all polymorphs are close to the ideal chair configuration, which would display an asymmetry parameter equal to zero. Each ring has three pseudo mirror planes (Figure 10). The calculated asymmetry parameter ΔC_s_ values for each of them are listed for comparison in Table 7. For a ring, the three values of the asymmetry parameter ΔC_s_ have different values and it makes sense to define the average value of the asymmetry parameter as the sum of the asymmetry parameters divided by three.

The closest value to the ideal geometry is found in ring B of Drost 3, molecule A (ΔC_s_ = 0.3), and, from the data listed in Table 6, it is concluded that, overall, in all structures, the asymmetry parameter ΔC_s_ has the smallest values in ring A, while greater are present in C rings.

**Figure 11:**
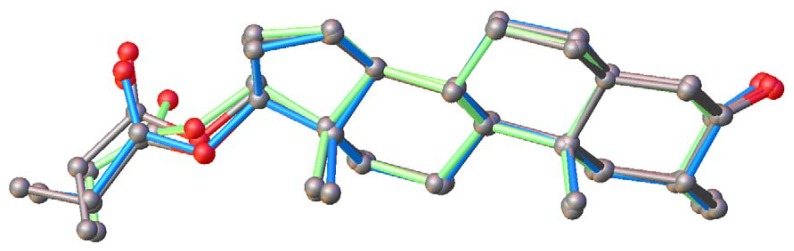
The overlay of the four molecular configurations: Drost 1-green, Drost 2-blue, and Drost 3 (A and B)-gray.

The maximal torsional parameter, τ_m_ (Table 8), in the studied compounds, was found to be relatively constant and close to 47°, which is a common value found in all D steroid rings [53] and other androstane derivatives [54]. Overlapping Drost 1, Drost 2, and the two molecules in the corresponding asymmetric unit of Drost 3, it is observed that A, B, C, and D rings overlap quite well, while the largest differences are observed in the propanoic acid terminals (Figure 11).

## 3. Materials and Methods

### 3.1. Materials

White crystalline powder of drostanolone propionate was received from the Chinese company Wuhan Shu Mai Technology Co. Ltd. (Wuhan, China).

### 3.2. Crystal Growth

Needle-shaped suitable single crystal for X-ray experiments were obtained for Drost 2 in ethanol solution by a slow evaporation method and plate-like crystals from acetone solution for Drost 3. No suitable single crystals were obtained for the starting compound known as Drost 1.

### 3.3. X-ray Powder Diffraction (XRPD)

X-ray powder diffraction pattern of Drost 1 was recorded with monochromatic radiation (CuKα1 radiation) obtained using a germanium monochromator on a Brucker D8 Advance diffractometer (tube operating at 40 kV, 40 mA), equipped with a LYNXEYE detector. The sample was scanned in the range 2θ = 5.5–40° with a step of 0.005 and 3 s/step.

### 3.4. Single Crystal X-ray Diffraction

Suitable single crystals of Drost 2 and Drost 3 were selected and mounted on a SuperNova diffractometer goniometer. The diffractometer was equipped with dual micro-sources, Eos CCD detector, with the experimental data being collected using CuKα radiation. Data collection and data reduction was performed with CrysAllis PRO software [55]. The crystal structures of both polymorphs were solved with Olex2 software [56] by Intrinsic Phasing method with ShelXT [57] structure solution program for Drost 2, while Drost 3 was solved using Direct Methods with SHELXS [58], which are both refined with the ShelXL [59] refinement package using Least Squares minimisation.

All non-hydrogenoid atoms were localized in the Fourier difference map and refined anisotropically with the displacement isotropic parameter U_iso_(H) = 1.2U_eq_(C) for all CH, CH_2_ groups and 1.5U_eq_(C) for all CH_3_ groups. Hydrogen atoms were placed in idealised positions and treated as riding as follows: ternary CH refined with riding coordinates (C-H = 0.98 Å), secondary CH_2_ refined with riding coordinates (C-H = 0.97 Å), and idealized CH_3_ methyl groups refined as a rotating group (C-H = 0.96 Å).

### 3.5. Evaluation of Intermolecular Interactions by Hirshfeld Surfaces and Fingerprint Plots

The analysis of three-dimensional Hirshfeld surfaces mapped with the d_norm_ function [60] offers the possibility to compare intermolecular contacts based on van der Waals radii in an interactive way with a red, white, and blue color mapping on the surface [61]. Hirschfeld surfaces and fingerprint plots are tools used in order to explore intermolecular interactions in polymorphs [62] and were generated in Crystal Explorer17 [63] software. The software uses CIF files as input and, during the computation, the C-H bond lengths are moved to the well-known standard distances determined by neutron diffraction (C-H = 1.083 Å). The d_norm_ function can be expressed as Equation (2), where d_i_ is considered the distance from the surface to the atom inside the surface, while d_e_ represents the distance from the surface toward the exterior atom and the van der Waals radii for both atoms inside and outside the surface.

(2) $$d_{norm}=\frac{d_{i}-r_{i}^{vdW}}{r_{i}^{vdW}}\;+\frac{d_{e}-r_{e}^{vdW}}{r_{e}^{vdW}}$$

### 3.6. Lattice Energy Evaluation by the CLP Model

The crystal lattice energy was calculated using the Coulomb-London–Pauli approximation, developed by Gavezotti and implemented in CLP software [64] as a sum of the following terms (Equation (3)), where I and j represent pairs of atoms belonging to two different molecules.

(3) $$E_{ij}=1/(4\pi \varepsilon_{0})\left(q_{i}q_{j}\right)R_{ij}^{-1}-F_{P}P_{ij}R_{ij}^{-4}-F_{D}D_{ij}R_{ij}^{-6}+F_{R}T_{ij}R_{ij}^{-12}$$

(4) $$q_{i}=F_{Q}q_{i}^{0}$$

In Equation (3), the first term is the Coulombian energy, which is treated according to Coulomb’s law. The second term is the polarization energy and is treated in the approximation of a linear dipole and depends on the inverse fourth power of distance. The third term represents the dispersion term and is approximated by the inverse of the distance to the sixth power. The last term represents the repulsion energy and is considered as a modulation of the overlapping wave function and depends on the distance between atoms at the twelfth-power. The *F_P_*, *F_D_*, *F_R_* coefficients in Equation (3) are empirical scale parameters and the *P_ij_*, *D_ij_*, and *T_ij_* coefficients are dependent on the vicinity of the atom in the molecule. $F_{Q}$ coefficient is included in Equation (4), $q_{i}$ being the rescaled net charge population on atom *i*, and $q_{i}^{0}$ is the charge in each atomic basin. 

### 3.7. Evaluation of Intermolecular Interactions by First Principal Methods

The intermolecular interaction energies between the drostanolone monomers were computed considering the DF-LMP2 method and using the Def2-TZVP [40] basis set as implemented in the Molpro program package [41]. To compute lattice energies for the three crystal configurations [34], the density-functional tight-binding (DFTB) model in its self-consistent charge corrected variant (SCC-DFTB) [32] implemented in the DFTB+ code [33] was applied. The sSAPT0 energy values were computed using the PSI4 software [65]. 

### 3.8. Conformational Analysis

**Figure 12:**
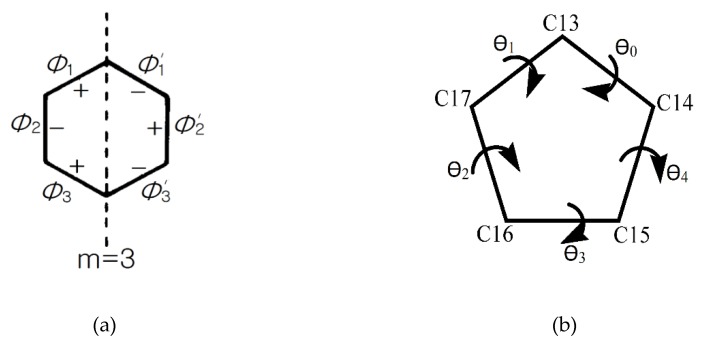
Torsion angles related by mirror planes considered for ΔC_s_ display opposite signs (**a**) torsional angles related to the angle of pseudorotion P and the maximum angle of torsion τ_m_ in five membered rings (**b**).

The conformational analysis of the A, B, and C steroid rings has been accomplished based on the asymmetry parameter ΔC_s_, which is defined in Equation (5) [46], where $\phi_{i}$ and $\phi_{i^{\prime }}$ represent the symmetry related torsion angles and m represents the pair numbers of torsion angles (Figure 12a). They have alternate opposite signs. For steroids, the torsion angle $\theta_{0}$ is considered as the angle corresponding to the common side between C and D rings.

The puckering in the five membered D rings of the steroid skeleton can be characterized by two distinct parameters: phase angle of pseudorotation, P defined in Equation (6) and the maximum torsion angle τ_m_ defined as Equation (7) and were derived by C. Altona and M. Sundaralingam [66]. In Equation (6), the torsion angles $\theta_{0}$, $\theta_{1}$, *θ*_2_, *θ*_3_, and *θ*_4_ related to the five-membered D ring are considered as shown in Figure 12b. *τ_m_* can be roughly approximated as the angle between the C13-C14-C17 and C14-C15-C16-C17 planes.

(5) $$\Delta C_{ѕ}=\sqrt{\frac{\sum_{i=1}^{m}{\left(\phi_{i}+\phi_{i\prime }\right)}^{2}}{m}}$$

(6) $$tan\;P=\frac{\left(\theta_{2}+\theta_{4}\right)-\left(\theta_{1}+\theta_{3}\right)}{2\theta_{0}\left(sin\;36+sin\;72\right)}$$

(7) $$\tau_{m}=\frac{\theta_{0}}{\cos P}$$

## 4. Conclusions

Starting from drostanolone propionate, two more polymorphs were obtained by recrystallization. The crystal structure for the starting compound was determined by X-ray powder diffraction using Parallel Tempering and refined by the Rietveld method, while, for two single crystals, the structures were determined by single crystal X-ray diffraction. The steroid skeletons were found to be very similar in all three polymorphs, with the A, B, and C rings depicting chair geometries and D rings envelope conformations. The most noticeable difference in molecular configurations were found to be in the propanoic acid terminals. Based on the calculation of the lattice energies with the CLP method, it was concluded that polymorphs obtained by recrystallization have almost the same lattice energies with respect to the starting compound. Hirschfeld surfaces analysis proved that the aggregates are held by combinations of C-H…O hydrogen bonds and by weak C-H…H-C contacts. The evaluation of the fingerprint plots showed that the interactions in the crystal structures are dominated by dispersive H...H contact interactions, followed by O....H/H...O, while C...H/H...C and O...C/C...O have a negligible contribution. Theoretical calculations based on the first principle quantum theory have demonstrated that the interaction between the unit cells is dominated by the dispersion type intermolecular forces. 

## Figures and Tables

**Table 1:** Single crystal X-ray experimental details.

Compound Name	Drost 2 (Ethanol)	Drost 3 (Acetone)
Empirical formula	C_23_H_36_O_3_	C_23_H_36_O_3_
Formula weight	360.52	360.52
Temperature/K	293(2)	293(2)
Crystal system	monoclinic	monoclinic
Space group	P2_1_	I2
a/Å	11.2322(5)	11.8778(5)
b/Å	7.4380(2)	7.4245(3)
c/Å	12.5035(5)	48. 6370(17)
α/°	90	90
β/°	93.647(2)	96. 668(4)
γ/°	90	90
Volume/Å^3^	1042.49(4)	4260.1(3)
Z	2	8
ρ_calc_g/cm^3^	1.149	1.124
μ/mm^−1^	0.576	0.564
F(000)	396.0	1584.0
Radiation	CuKα (λ = 1.54184)	CuKα (λ = 1.54184)
2Θ range for data collection/°	7.064 to 140.916	7.32 to 144.44
Index ranges	−13 ≤ h ≤ 13, −9 ≤ k ≤ 8, −15 ≤ l ≤ 15	−14 ≤ h ≤ 14, −8 ≤ k ≤ 8, −59 ≤ l ≤ 59
Reflections collected	13976	30229
Independent reflections	3924 [R_int_ = 0.0356, R_sigma_ = 0.0232]	8013 [R_int_ = 0.0195, R_sigma_ = 0.0146]
Data/restraints/parameters	3924/1/239	8013/2/477
Goodness-of-fit on F^2^	1.017	1.036
Final R indexes [I > = 2σ (I)]	R_1_ = 0.0446, wR_2_ = 0.1266	R_1_ = 0.0465, wR_2_ = 0.1317 8
Final R indexes [all data]	R_1_ = 0.0475, wR_2_ = 0.1315	R_1_ = 0.0566, wR_2_ = 0.1463
Largest diff. peak/hole/e Å^−3^	0.16/−0.16	0.20/−0.13
Flack parameter	0.10(10)	0.06(6)
CCDC No.^*^	1956813	1956814

^*^ For details, see the description given for the Supplementary Materials.

**Table 2:** Drost 1 crystal data^*^.

Chemical Formula	C_23_H_36_O_3_
Formula weight (g/mol)	360.53
Crystal system	orthorhombic
Space group	P 2_1_ 2_1_ 2_1_ (19)
Z	4
a (Å)	27.2532(19)
b (Å)	12.0731(8)
c (Å)	6.4161(4)
V (Å^3^)	2111.09
R_wp_ (%)	5.64
CCDC No.	1956812

^*^ For details, see the description given for the Supplementary Materials.

**Table 3:** Intermolecular contacts with distances shorter than the sum of van der Waals radii (Å and Degree).

Structure	D-H...A	d(D-H)	d(H...A)	d(D...A)	<(DHA)	Label	Fig.
Drost 1	C6-H6A...O3	0.97	2.715(3)	3.647(2)	161.43(1)	1 (White spot)	7
	C19-H19A...H5-C5	0.96/0.98	2.373(7)	3.907(6)	146.15(7)/138.6(8)	2 (White spot)	7
Drost 2	C19-H19B...O3	0.96	2.687(5)	3.622(8)	164.54(2)	1 (Faint-red spot)	7
	C12-H12A...H5-C5	0.97/0.98	2.341(1)	4.047(7)	136.2(4)/152.04(8)	2 (Faint-red spot)	7
Drost 3 mol. A	C5B-H5B...O1A	0.98	2.537(8)	3.460(11)	157.09(9)	1 (Intense-red spot)	7
	C1B-H1BB...O1A	0.97	2.720(6)	3.554(7)	144.43(4)	1 (Intense-red spot)	7
	C4A-H4AA...O1B	0.97	2.510(8)	3.459(9)	166.38(6)	2 (Intense red spot)	7
	C22A-H22C...C20A	0.96	3.077(1)	4.031(1)	174.06(3)	4 (White spot)	7
Drost 3 mol. B	C5B-H5B...O1A	0.98	2.537(8)	3.460(11)	157.09(9)	1 (Intense red spot)	7
	C1B-H1BB...O1A	0.97	2.720(6)	3.554(7)	144.43(4)	1 (Intense red spot)	7
	C4A-H4AA...O1B	0.97	2.510(8)	3.459(9)	166.38(6)	2 (Intense red spot)	7
	C21B-H21D...H22E-C22B	0.97/0.96	2.366(7)	4.086(9)	141.02(8)	3 (White spot)	7
	C4B-H4BA...O1B	0.97	2.665(8)	3.524(6)	118.68(2)	4 (White spot)	7

**Table 4:** Various contributions to the Hirshfeld surface for different interactions.

Structure	H...H	O...H/H...O	C...H/H...C	C...O/O...C	O…O	C…C
Drost 1	83.2%	16.0%	0.5%	0.3%	-	-
Drost 2	83.3%	14.6%	0.9%	0.5%	0.8%	-
Drost 3 Mol. A	83.4%	14.3%	1.1%	0.6%	0.6%	-
Drost 3 Mol. B	82.0%	16.7%	0.5%	0.5%	0.2%	0.1%
Drost 3 overall	85.2%	12.9%	0.9%	0.6%	0.4%	-

**Table 5:** **a.** CLP energy components (E_coul_—Coulombic, E_pol_—polarization, E_disp_—dispersion, E_att_—attraction (the sum of Coulombic, polarization, dispersion terms), E_rep_—repulsion terms) and lattice energies (E_latt_—CLP crystal lattice energy) for the three crystal conformations (all energy values are given in kJ/mol). **b.** The lattice energies and their dispersion energy components (in kJ/mol) for the three crystal configurations of the drostanolone propionate molecule computed at the self-consistent charge density functional tight-binding (SCC-DFTB) level of theory.

**a**						
**Structure**	**E_coul_**	**E_pol_**	**E_disp_**	**E_att_**	**E_rep_**	**E_latt_ (kJ/mol)**
Drost 1	−16.0	−54.1	−130.4	−200.5	44.2	−156.3
Drost 2	−10.3	−56.5	−139.4	−206.2	48.7	−159.2
Drost 3	−13.0	−55.2	−134.7	−202.9	51.4	−151.6
**b**						
**Structure**	**E_disp_ (*N*^∞^)**	**E_latt_ (*N*^∞^)**				
Drost 1	−125.9	−142.6				
Drost 2	−138.3	−151.7				
Drost 3	−119.7	−137.7				

**Table 6:** The intermolecular energy values and their different energy components computed at sSAPT0 (E_electr_—electrostatic, E_exch_—exchange, E_ind_—induction and E_disp_—dispersion) as well as the intermolecular energy values obtained at DF-LMP2 levels of theory for the five dimer configurations taken from the Drost2 crystal conformations (all energy values are given in kJ/mol).

Structure	E_electr_	E_exch_	E_ind_	E_disp_	E_sSAPT0_	E_DF-LMP2_
Dim *a)*	−3.50	+18.25	−4.07	−39.45	−28.77	−21.67
Dim *b)*	−5.36	+7.68	−2.82	−19.97	−20.47	−15.37
Dim *c)*	−3.48	+11.79	−2.24	−30.06	−24.00	−18.08
Dim *d)*	−6.22	+3.54	−1.08	−7.24	−10.98	−8.25
Dim *e)*	−11.06	+8.39	−2.96	−12.94	−18.47	−14.13

**Table 7:** Calculated asymmetry parameter values in studied compounds.

	Mirror Planes	Drost 1	Drost 2	Drost 3-Mol A	Drost 3-Mol B
Ring A	ΔC_s_ (C3–C10)	1.22	2.72	0.69	2.43
	ΔC_s_ (C4–C1)	1.12	0.90	2.93	2.51
	ΔC_s_ (C5–C2)	1.27	1.85	3.41	4.49
	Average ΔC_s_	1.20	1.82	2.34	3.14
Ring B	ΔC_s_ (C5–C8)	1.46	2.67	3.43	2.28
	ΔC_s_ (C6–C9)	4.50	3.37	0.30	4.33
	ΔC_s_ (C7–C10)	3.73	0.75	3.14	6.13
	Average ΔC_s_	3.23	2.27	2.29	4.24
Ring C	ΔC_s_ (C9–C13)	3.49	2.00	2.26	2.35
	ΔC_s_ (C8–C12)	4.21	8.62	6.94	3.49
	ΔC_s_ (C14–C11)	0.84	6.86	8.79	5.62
	Average ΔC_s_	2.85	5.83	5.99	3.82

**Table 8:** Pseudo-rotation and maximum torsion angles in D rings (Degree).

*Structure*	*Drost 1*	*Drost 2*	*Drost 3*	
			*Mol. A*	*Mol. B*
*P*	*7.23*	*11.19*	*10.7*	*6.95*
*τ_m_*	*46.26*	*46.94*	*46.72*	*46.86*

## Supplementary Materials

The CIF-files have been deposited with the Cambridge Crystallographic Data Centre with the 1956812-1956814 deposition numbers as follows: 1956812 for Drost 1, 1956813 for Drost 2 and 1956814 for Drost 3. Copies of them can be obtained free of charge on written application to CCDC, 12 Union Road, Cambridge, CB2 1EZ, UK (Fax: +44-1223-336033); on request via e-mail to deposit@ccdc.cam.uk or by access to http://www.ccdc.cam.ac.uk.
